# Analysis of trend in mortality due to HIV/AIDS-defining and non-HIV/AIDS defining illnesses according to sociodemographic characteristics, by Federative Unit and Brazil, 2000-2018

**DOI:** 10.1590/S2237-96222022000200021

**Published:** 2022-10-03

**Authors:** Ana Paula da Cunha, Marly Marques da Cruz

**Affiliations:** 1 Fundação Oswaldo Cruz, Escola Nacional de Saúde Pública Sergio Arouca, Rio de Janeiro, RJ, Brazil

**Keywords:** Mortality, HIV, Acquired Immunodeficiency Syndrome, Time Series Studies

## Abstract

**Objective:**

To analyze the temporal trend of mortality rate due to HIV/AIDS defining and non-HIV/AIDS defining illnesses in Brazil between 2000 and 2018.

**Methods:**

This was an ecological time series study, using data from the Mortality Information System, in Brazil and the Federative Units. Trend analysis was performed by means of Prais-Winsten regression model, according to overall mortality rate, sex, age group, marital status and race/skin color.

**Results:**

A total of 237,435 deaths were recorded in the period. In the country, defining illnesses showed higher rates (7.4 to 4.4 deaths/100,000 inhabitants in the period) than those observed among non-defining diseases (0.4 to 0.8 death/100,000 inhabitants in the period). It could be seen a decrease in overall mortality due to defining diseases (-6.3%; 95%CI -8.8;-3.8); while it increased due to non-defining diseases (11.0%; 95%CI 6.5;15.7).

**Conclusion:**

There was a change in HIV/AIDS mortality profile over the years, with a decrease in deaths due to HIV/AIDS-defining diseases.

**Study contributions**

**Main results**

This study showed that mortality due to non-HIV/AIDS defining illnesses has increased. Despite this scenario, it can be seen that the rates for HIV/AIDS-defining illnesses are still expressive.

**Implications for services**

The knowledge of the profile of mortality due to HIV/AIDS according to defining and non-defining illnesses contributes to the management of service conduct.

**Perspectives**

It is expected that the findings of this study will contribute to the strategies for services that provide care for people living with HIV/AIDS, in order to reduce deaths from the disease.

## INTRODUCTION

In 1996, universal, free of charge antiretroviral therapy (ART) was made available by the Brazilian National Health System (SUS).^1^ ART increased survival and improved quality of life among people living with human immunodeficiency virus (HIV) and those with manifestation of acquired immunodeficiency syndrome (AIDS).^2^

This treatment led to the occurrence of deaths from age-related diseases or long-term ART use, classified as non-HIV/AIDS defining illnesses, such as arterial hypertension, diabetes *mellitus*, heart diseases, neoplasms, kidney diseases among others. Thus, there was a decline in deaths from diseases associated with immunodeficiency that, until then, enabled the emergence of opportunistic illnesses, which are HIV/AIDS-defining illnesses, characterized by the occurrence of some specific diseases among people living with HIV/AIDS (PLHIV) who are immunosuppressed due to the evolution of HIV infection and the manifestation of AIDS, such as pneumonia and tuberculosis.^2^ ^,^ ^3^

Changing patterns of deaths from HIV/AIDS has given the health condition the status of chronic disease, although its defining illnesses still remain leading causes of death.^2^

Several factors may contribute to mortality due to HIV/AIDS in Brazil, such as sociodemographic characteristics, unfavorable living conditions and clinical issues, making it complex to define what primarily determines death from the disease.^4^ ^,^ ^5^

Worldwide, there was a 39% decrease in the number of deaths due to HIV/AIDS recorded between 2010 (1,100,000 deaths) and 2019 (780,000 deaths), being this decrease attributed to adherence to drug treatment for PLHIV.^6^

In Brazil, it could be seen a reduction in mortality due to HIV/AIDS after the distribution of ART, from approximately 10 deaths/100,000 inhabitants in 1995 to about 7 deaths/100,000 inhabitants, in 2000, four years after the implementation of the policy for universal and free of charge distribution of ART.^7^ Currently, in certain Federative Units, mortality rates are higher than those found in the country,^7^ a fact that may be related to a weakening of the Brazilian initiative in the response to HIV and disease control, in addition to a context of regional inequalities.^6^ ^,^ ^8^

Knowledge of the mortality profile based on sociodemographic characteristics and according to the type (defining and non-defining illnesses) is necessary. In the literature, most studies focus on the occurrence of diseases among PLHIV^3^ rather than a comparison between defining and non-defining illnesses, which is precisely this research proposal, and the purpose of which is to contribute to the understanding of deaths due to HIV/AIDS based on the type and sociodemographic factors. Moreover, this research can support the direction for public policies, incorporating the population profile according to defining and non-defining illnesses of infection and condition.

The aim of this study was to analyze the temporal trend of mortality rate due to HIV/AIDS defining and non-HIV/AIDS defining illnesses according to sociodemographic characteristics in Brazil, between 2000 and 2018.

## METHODS

This was an ecological time series study of mortality rates due to HIV/AIDS defining and non-HIV/AIDS defining illnesses. The units of analysis were comprised of FUs and the country, and all deaths from HIV/AIDS recorded between 2000 and 2018 were taken into consideration.

Brazil has an extensive territorial area, with 8,510,345.538 km^2^ and a population of 213,317,639 people, corresponding to a population density of 22.43 inhabitants/km^2^, with 99.7% of the population aged between 6 and 14 at school age, while it has an illiteracy rate of 6.6% among those aged 15 years and over. The fertility rate in the country is 1.76 child per woman, and the gross domestic product (GDP) per capita was BRL 35,161.70. The Gini index in Brazil was 0.543 in 2019, while the human development index (HDI) was 0.765 that same year.

The country is divided into five macro-regions (North, Northeast, Midwest, South and Southeast regions), and subdivided into 26 states and the Federal District. The North region comprises 45% of the national territory, has a population of 18.6 million inhabitants, GDP corresponding to 5.3% of the national GDP, HDI of 0.730 and Gini index, 0.538. The Northeast region has an area of 1,554,291.744 km^2^, a population of 57 million inhabitants, GDP of BRL 1,004,828 million, HDI 0.710, and Gini index, 0.559. The Midwest has an area of 1,606,403.506 km², a population of 16,085,885 inhabitants, GDP, BRL 542.632 billion; HDI of 0.789; and Gini index, 0.503. The Southeast region corresponds to an area of 924,620.678 km², with a population of 87,711,946 inhabitants, GDP of BRL 2,295,690 million, HDI of 0.794 and Gini index, 0.525. The South region comprises an area of 7% of the Brazilian territory and had a population of 29,975,984 inhabitants, GDP of BRL 1.12 trillion, HDI of 0.756 and Gini index of 0.467. All these data are related to the year 2019.

This study used microdata from the Mortality Information System (SIM, as per the Brazilian acronym), via the Brazilian National Health System Information Technology Department (DATASUS, as per the Brazilian acronym) website in January 2022,^9^ in order to make it possible to classify deaths from defining and non-defining illnesses. It included all deaths registered in the International Statistical Classification of Diseases and Related Health Problems - 10^th^ Revision (ICD-10), between the items B20 and B24, related to the group “Disease caused by the human immunodeficiency virus” in the underlying cause or in lines A, B, C, D and II of the Death Certificate (DC).

The classification of a death as HIV/AIDS-defining illness included all deaths presented in the classification of HIV/AIDS-defining illnesses of the Coding Causes of Death in HIV Protocol, published by the Centers for Disease Control and Prevention (CDC) of the United States (Box 1).^10^ With regard to non-defining illnesses, all those that were not in the list of defining illnesses were taken into consideration (Box 1).

Deaths were analyzed and classified as “death from defining illness” or “death from non-defining illness”, based on ICD-10 items registered in lines A, B, C, D and II of the DC. Deaths whose underlying cause presented ICD were identified, however, lines A, B, C, D and II did not have a register in the Classification. In these cases, the ICDs registered were those classified between items B20 and B24 of ICD-10 as HIV/AIDS - defining illnesses, because they are ICDs that characterize the occurrence of death from opportunistic infections.

Mortality rates due to HIV/AIDS defining and non-HIV/AIDS defining illnesses per 100,000 inhabitants were standardized using the direct method, and the Brazilian population was defined as a standard population.^11^ Standardization using the direct method aims at adjusting the effect of age on the general mortality rate (GMR), taking into consideration a standard population, with known age distribution, from which we can identify the weights known by the proportion of people in each age group, which will be applied to age-specific mortality rates.^1^ Regarding the direct method, GMRs by age groups of a population are applied to the population size of the standard population, making it possible to identify the expected deaths in each age group and, from the division of the total number of expected deaths by the standard population, calculate the standardized GMR.^11^

Mortality rates considered, as numerator, deaths from defining/non-defining illnesses, and as denominator, the population in the period. This calculation was performed according to the sociodemographic characteristics aforementioned.

The standardizations was made for general mortality, sex (female; male), marital status (married; unmarried) and race/skin color (White; Black). The analysis by race/skin color comprised the White and Black categories, the latter resulting from the aggregation of Black and Brown categories, while for the White race/skin color no aggregation was necessary since it was a single category. The aggregation of Blacks and Browns as Blacks was performed to enable the analysis of the Afro-descendant population in a unified way.

The variable “marital status” was also categorized, between married and unmarried. This aggregation was performed by the categories that represented the same meaning, married and unmarried, namely: single; married; widower; legally separated; in consensual union. For the category “married”, the categories “married” and “legally separated” were aggregated, while for “unmarried”, the stratifications “single”, “widower” and “legally separated” were aggregated.

Mortality rates were also calculated according to age group, in years: 0 to 14; 15 to 29; 30 to 59; 60 or over.

Population data, necessary to calculate mortality rates, were retrieved from the DATASUS and Instituto Brasileiro de Geografia e Estatística (IBGE) websites in November 2021.^8^ Regarding the calculation of the general mortality rates and age group, the population estimates available at the DATASUS website were used.^8^

With regard to the populations, according to race/skin color and marital status, it was necessary to extract data from IBGE. These data were accessed via the Application Programming Interface (API) of the Automatic Recovery System (SIDRA, as per the Brazilian acronym), using the SidraR package via the Rstudio statistical program. These populations are available for census years 2000 and 2010, so projections were made for the intercensal years up to 2018. The projections were calculated using the geometry projection method, which considers population growth to be constant.

Prais-Winsten regression model was used for trend analysis.^12^ Independent variables (X) refer to the years when the deaths occurred, and dependent variables (Y) correspond to the mortality rates. This model is applied in order to correct serial autocorrelation in time series, and it is necessary to use the Durbin-Watson test, in which the value of the test is measured from a scale ranging from 0 to 4. Values close to zero indicate the existence of maximum positive autocorrelation. When the values are close to 4, the serial autocorrelation is negative. However, if the Durbin-Watson value is close to 2, there is no serial autocorrelation.^12^

After the analysis of serial autocorrelation, the logarithmic transformation of (Y) values was performed in order to reduce the heterogeneity of the variance of residuals of the model. Subsequently, the Prais-Winsten regression model was used in order to estimate the *b1* values of mortality rates. The *b1* values of each of the rates were applied to the following formula for the calculation of the annual percentage change (APC):

$$APC:\;[-1+e^{b1}]\;*100\%$$

Positive APC indicates an upward trend, while the negative corresponds to a decreasing trend; the series is called stationary when there is no significant difference between its value and zero.^12^

Finally, the 95% confidence intervals (95%CI) of the study measurements were calculated using the following formula:

$$95\%CI\;=\;[-1+10^{bminimum}]\;*100\%;\;[-1+10^{bmaximum}]\;*100\%$$

The minimum and maximum values of *b* were identified from the 95%CI parameters generated by the statistical analysis software and applied in the formula, with the minimum value of *b* corresponding to the minimum point of CI, and the maximum value of *b* corresponding to the maximum point of CI.

The significance level considered was 5% in the Prais-Winsten model, for trend analysis. The steps of data organization, rate calculation, trend analysis and graphs were developed using the RStudio software, version 4.0.2.

This study was performed using publicly available secondary data, and did not involve interactions with humans. However, the project was submitted to the Research Ethics Committee of the Escola Nacional de Saúde Pública Sergio Arouca/Fundação Oswaldo Cruz (CEP/ENSP/Fiocruz), and was approved, Opinion No. 16, issued on November 23, 2020.

## RESULTS

**Figure 1:**
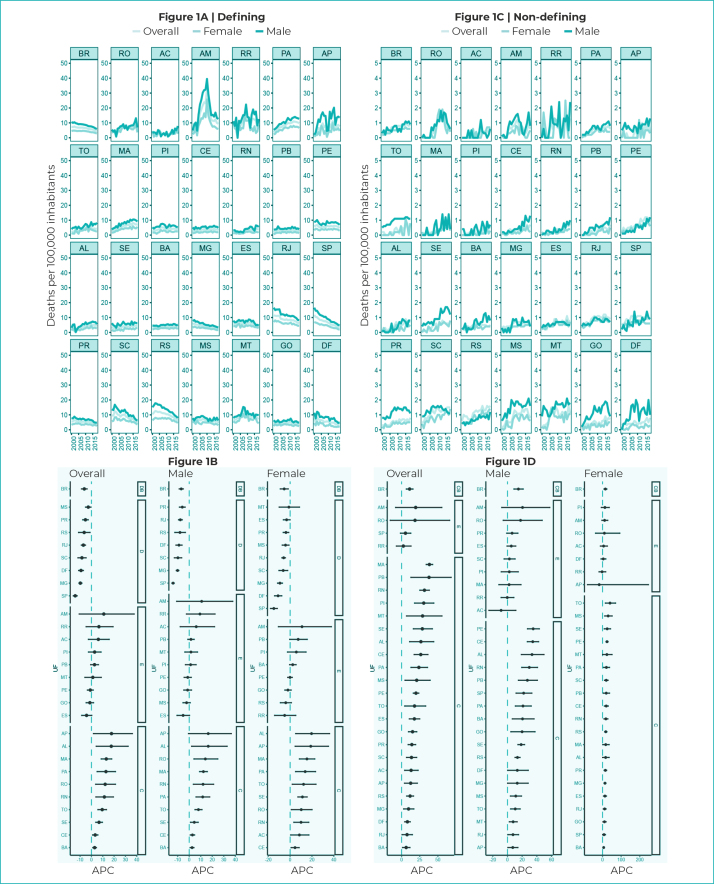
- Mortality rates and 95% confidence intervals with trends in HIV/AIDS-defining and non-HIV/AIDS illnesses, according to general mortality and by sex, Federative Units and Brazil, 2000-2018

Legend: DB - decreasing trend in Brazil; SB - stationary trend in Brazil; IB - increasing trend in Brazil; D - decreasing trend; S - stationary trend; I - increasing trend; APC - Annual Percentage Change.

A total of 237,435 deaths from HIV/AIDS were recorded between 2000 and 2018. General mortality rates due to HIV/AIDS-defining illnesses ranged from 7.4 death/100,000 inhabitants, in 2000, to 4.4 death/100,000 inhabitants, in 2018. For non- HIV/AIDS defining illnesses, general mortality rates ranged from 0.4 deaths/100,000 inhabitants, in 2000, to 0.8 deaths/100,000 inhabitants, 2018 (Figure 1A).

**Box 1:** - Correspondence of the International Statistical Classification of Diseases and Related Health Problems - 10^th^ Revision (ICD-10) by defining diseases established by the Centers for Disease Control and Prevention (CDC) of the United States

ICD-10 - Description			
A02.1 Salmonella sepsis	A31.1 Cutaneous mycobacterial infection	D75.2 Essential thrombocytosis	A17.0 Tuberculous meningitis
A07.3 Isosporiasis	A31.8 Other mycobacterial infections	E43 Unspecified severe protein-calorie malnutrition	A17.1 Meningeal tuberculoma
A09 Infectious gastroenteritis and colitis, unspecified	A31.9 Mycobacterial infection, unspecified	E43 Unspecified severe protein-calorie malnutrition	A17.8 Other tuberculosis of nervous system
A09 Infectious gastroenteritis and colitis, unspecified	A40.3 Sepsis due to Streptococcus pneumoniae	E44.0 Moderate protein-calorie malnutrition	A17.9 Tuberculosis of nervous system, unspecified
A15.0 Tuberculosis of lung, confirmed by sputum microscopy with or without culture	A68.9 Relapsing fever, unspecified	E44.1 Mild protein-calorie malnutrition	A18.0 Tuberculosis of bones and joints
A15.1 Tuberculosis of lung/confirmed by culture only	A81.2 Progressive multifocal leukoencephalopathy	E46 Unspecified protein-calorie malnutrition	A18.1 Tuberculosis of genitourinary system
A15.2 Tuberculosis of lung, confirmed histologically	B01.2 Varicella pneumonia	E46 Unspecified protein-calorie malnutrition	A18.2 Tuberculous peripheral lymphadenopathy
A15.3 Tuberculosis of lung, confirmed by unspecified means	B02.0 Zoster encephalitis	J11.0 Influenza due to unidentified influenza virus with unspecified type of pneumonia	A18.3 Tuberculosis of intestines, peritoneum glands
A15.4 Tuberculosis of intrathoracic lymph nodes, confirmed bacteriologically and histologically	B02.1 Zoster meningitis	J12.0 Adenoviral pneumonia	A18.4 Tuberculosis of skin and subcutaneous tissue
A15.5 Tuberculosis of larynx, trachea and bronchus, confirmed bacteriologically and histologically.	B02.2 Zoster with other nervous system involvement	J12.1 Respiratory syncytial virus pneumonia	A18.5 Tuberculosis of eye
A15.6 Tuberculosis pleurisy, confirmed bacteriologically and histologically	B02.3 Zoster ocular disease	J64 Unspecified pneumoconiosis	A18.7 Tuberculosis of adrenal glands
A15.7 Primary respiratory tuberculosis, confirmed bacteriologically and histologically	B02.7 Disseminated zoster	J65 Pneumoconiosis associated with tuberculosis	A18.8 Tuberculosis of other specified organs
A15.8 Other respiratory tuberculosis, confirmed bacteriologically and histologically	B02.8 Zoster with other complications	J65 Pneumoconiosis associated with tuberculosis	A19.0 Acute miliary tuberculosis of a single specific site
A15.9 Respiratory tuberculosis unspecified, confirmed bacteriologically and histologically.	B02.9 Zoster without complications	J67.8 Hypersensitivity pneumonitis due to other organic dusts	A19.1 Acute miliary tuberculosis of multiple sites
A15 Respiratory tuberculosis, confirmed bacteriologically and histologically	B25.0 Cytomegaloviral pneumonitis	J68.0 Bronchitis and pneumonitis due to chemicals, gases, fumes and vapors	A19.2 Acute miliary tuberculosis, unspecified
A16.0 Tuberculosis of lung, bacteriologically e histologically negative	B25.1 Cytomegaloviral hepatitis	J69.0 Pneumonitis due to inhalation of food and vomit	A19.8 Other miliary tuberculosis
A16.1 Tuberculosis of lung, bacteriological and histological examination not done	B25.2 Cytomegaloviral pancreatitis	J69.8 Pneumonitis due to inhalation of other solids and liquids	A19.9 Miliary tuberculosis, unspecified
A16.2 Tuberculosis of lung, without mention of bacteriological or histological confirmation	B25.8 Other cytomegaloviral diseases	J85.1 Abscess of lung with pneumonia	A31.0 Pulmonary mycobacterial infection
A16.3 Tuberculosis of intrathoracic lymph nodes, without mention of bacteriological or histological confirmation	B25.9 Cytomegaloviral disease, unspecified	K59.1 Functional diarrhea	C85 Other specified and unspecified types of non-Hodgkin lymphoma
A16.4 Tuberculosis of larynx, trachea and bronchus, without mention of bacteriological or histological confirmation	B37.1 Pulmonary candidiasis	R05 Cough	D46.0 Refractory anemia without ring sideroblasts
A16.5 Tuberculosis pleurisy without mention of bacteriological or histological confirmation	B39.3 Disseminated histoplasmosis capsulati	R50.1 Relapsing fever	D46.4 Refractory anemia, unspecified
A16.7 Primary respiratory tuberculosis, without mention of bacteriological or histological confirmation	B39.4 Histoplasmosis capsulati, unspecified	R50 Fever of other and unknown origin	D50.0 Iron deficiency anemia secondary to blood loss
A16.8 Other respiratory tuberculosis, without mention of bacteriological. or histological confirmation	B39.5 Histoplasmosis duboisii	R64 Cachexia	D50.8 Other iron deficiency anemias
A16.9 Respiratory tuberculosis unspecified, without mention of bacteriological or histological confirmation	B39.9 Histoplasmosis, unspecified	J17.1 Pneumonia in diseases classified elsewhere	D50.9 Iron deficiency anemia, unspecified
D52.0 Dietary folate deficiency anemia	J17.2 Pneumonia in mycoses, classified elsewhere	D51.0 Vitamin B12 deficiency anemia due to intrinsic factor deficiency	C46.9 Kaposi’s sarcoma, unspecified
B45.1 Cerebral cryptococcosis	J17.3 Pneumonia in parasitic diseases, classified elsewhere	D51.1 Vitamin B12 deficiency anemia due to selective vitamin B12 malabsorption with proteinuria	C82.9 Follicular lymphoma, unspecified
B45.2 Cutaneous cryptococcosis	J18.0 Bronchopneumonia, unspecified organism	D51.9 Vitamin B12 deficiency anemia, unspecified	C83.8 Other non-follicular lymphoma
B45.7 Disseminated cryptococcosis	J18.1 Lobar pneumonia, unspecified organism	J15.0 Pneumonia due to Klebsiella pneumoniae	C83.9 Non-follicular (diffuse) lymphoma, unspecified
B45.8 Other forms of cryptococcosis	J18.2 Hypostatic pneumonia, unspecified organism	J15.1 Pneumonia due to pseudomonas	C85.7 Other specified types of non-Hodgkin lymphoma
B45.9 Cryptococcosis, unspecified	J18.8 Other pneumonia, unspecified organism	J16.0 Chlamydial pneumonia	C85.9 Non-Hodgkin lymphoma, unspecified
B58.2 Toxoplasma meningoencephalitis	J18.9 Pneumonia NE	J16.8 Pneumonia due to other specific infectious organisms	D69.4 Other primary thrombocytopenia
B95.3 Streptococcus pneumoniae as the cause of disease classified elsewhere	J18 Pneumonia, unspecified organism	J15.4 Pneumonia due to other streptococci	D69.5 Secondary thrombocytopenia
B96.0 M. pneumoniae as the cause disease classified elsewhere	J64 Unspecified pneumoconiosis	J15.5 Pneumonia due to Escherichia coli	D69.6 Thrombocytopenia, unspecified
B96.1 K. pneumoniae as the cause of disease classified elsewhere	D52.9 Folate deficiency anemia, unspecified	J15.6 Pneumonia due to other gram-negative bacteria	J15.2 Pneumonia due to Staphylococcus
C46.0 Kaposi’s sarcoma of skin	D53.0 Protein deficiency anemia	J15.7 Pneumonia due to Mycoplasma pneumoniae	J15.3 Pneumonia due to Streptococcus do group B
C46.1 Kaposi’s sarcomoma of soft tissue	D53.2 Scorbutic anemia	J15.8 Pneumonia due to other specified bacteria	J12.9 Viral pneumonia, unspecified
C46.2 Kaposi’s sarcoma of palate	D53.8 Other specified nutritional anemias	J15.9 Unspecified bacterial pneumonia	J13 Pneumonia due to Streptococcus pneumoniae
C46.3 Kaposi’s sarcoma of lymph nodes	D53.9 Nutritional anemia, unspecified	J12.2 Parainfluenza virus pneumonia	J14 Pneumonia due to Hemophilus infuenzae
C46.7 Kaposi’s sarcoma of other sites	D55.0 Anemia due to glucose-6-phosphate dehydrogenase deficiency	J12.8 Other viral pneumonia	D69.3 Immune thrombocytopenic purpura
C46.8 Kaposi’s sarcoma of multiple organs			

In Brazil, there was a decreasing trend in mortality due to defining illnesses for the general population (APC = -6.3%; 95%CI -8.8;-3.8), as well as for females (APC = -5.4%; 95%CI -9.0;-1.7) and male (APC = -6.9%; 95%CI -8.7;-5.1) in the analysis of non-HIV/AIDS defining illnesses.

However, an upward trend was found in the states of the North and Northeast regions, mainly for HIV/AIDS-defining illnesses (Figure 1B).

It could be seen an upward trend among non-HIV/AIDS defining illnesses in Brazil (APC = 11.0%; 95%CI 6.5;15.7) and in the states, with the exception of São Paulo (APC = 5.3%; 95%CI -1.6;12.7), Amazonas (APC = 19.4%; 95%CI 8.3;55.4) and Roraima (APC = 2.4%; 95%CI -7.2;13.0) (Figure 1D).

**Figure 2:**
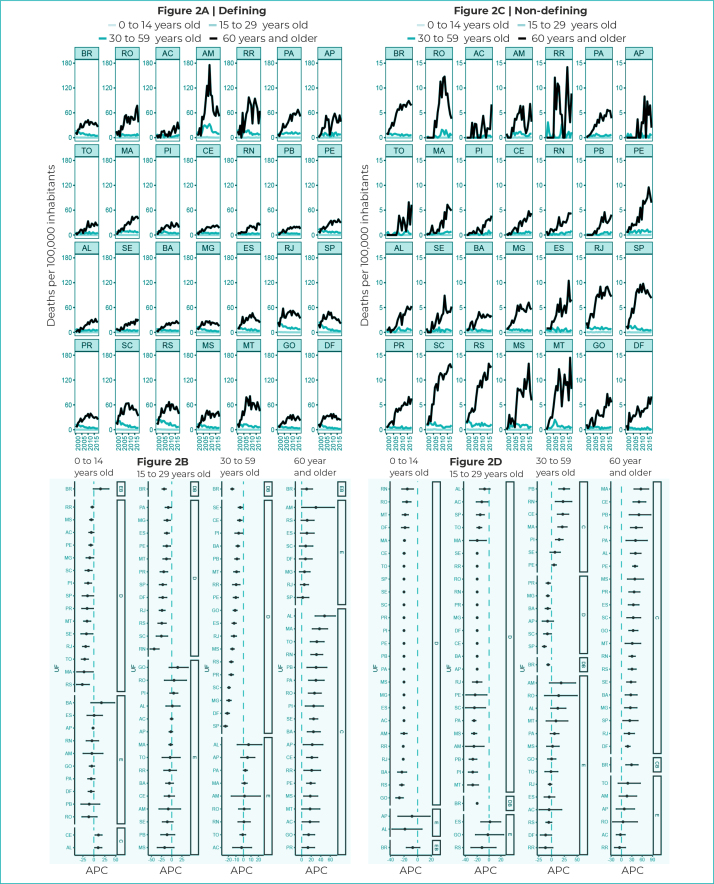
- Mortality rates and 95% confidence intervals with trends in HIV/AIDS-defining and non-HIV/AIDS defining illnesses, according to age group, Federative Units and Brazil, 2000-2018

Legend: DB - decreasing trend in Brazil; SB - stationary trend in Brazil; IB - increasing trend in Brazil; D - decreasing trend; S - stationary trend; I - increasing trend; APC - Annual Percentage Change.

With regard to mortality due to defining illnesses, according to age group, the rates were significant among those aged 60 years and over in the country (from 13.1 deaths in 2000 to 26.0 deaths/100,000 inhabitants in 2018), as well as among those aged 30 to 59 years (from 16.0 deaths in 2000 to 4.8 deaths/100,000 inhabitants in 2018) (Figure 2A). Rates related to defining illnesses in the 0 to 14 and 15 to 29 age groups showed close values, the lowest being close to 0.2 death/100,000 inhabitants (Figure 2A).

Trend in mortality due to defining illnesses showed that in the 0 to 14 age group, only Ceará (APC = 10.5%; 95%CI 2.9;-18.7) and Alagoas (APC = 9.8%; 95%CI 2.1;18.1) showed an upward trend in mortality rates due to defining illnesses (Figure 2B). It could be seen that in the 15 to 29 age group, there was no increasing trend in any units of analysis, a fact that was also observed in the 30 to 59 age group (Figure 2B).

Among those aged 60 years and older, there was an upward trend in most of the geographic units of analysis, with the exception of the country (APC = 10.4%; 95%CI-0.5;22.6) and the states of Amazonas (APC = 29.8%; 95%CI -0.1;68.5), Rio Grande do Sul (APC = 11.5%; 95%CI -1.1;25.9), Espírito Santo (APC = 10.9%; 95%CI -2.5;26.1), Santa Catarina (APC = 8.4%; 95%CI -4.7;23.3), Federal District (APC = 7.9%; 95%CI -4.6;21.9), Minas Gerais (APC = 5.9%; 95%CI -4.5;17.5), Rio de Janeiro (APC = 5.5%; 95%CI -2.7;-14.2) and São Paulo (APC = 2.1%; 95%CI -9.3;14.9), which showed a stationary trend (Figure 2B).

Regarding non-HIV/AIDS defining illnesses, taking into consideration the country as a whole, there was a mostly decreasing trend in the 15 to 29 (APC = -20.1%; 95%CI -20.7;-19.5) and 30 to 59 (APC = -6.6%; 95%CI -9.1;-3.9) age groups. However, for those aged 60 years or over, there was an upward trend in most states and in Brazil (APC = 29.1%; 95%CI 12.6;-48.0) (Figure 2D).

**Figure 3:**
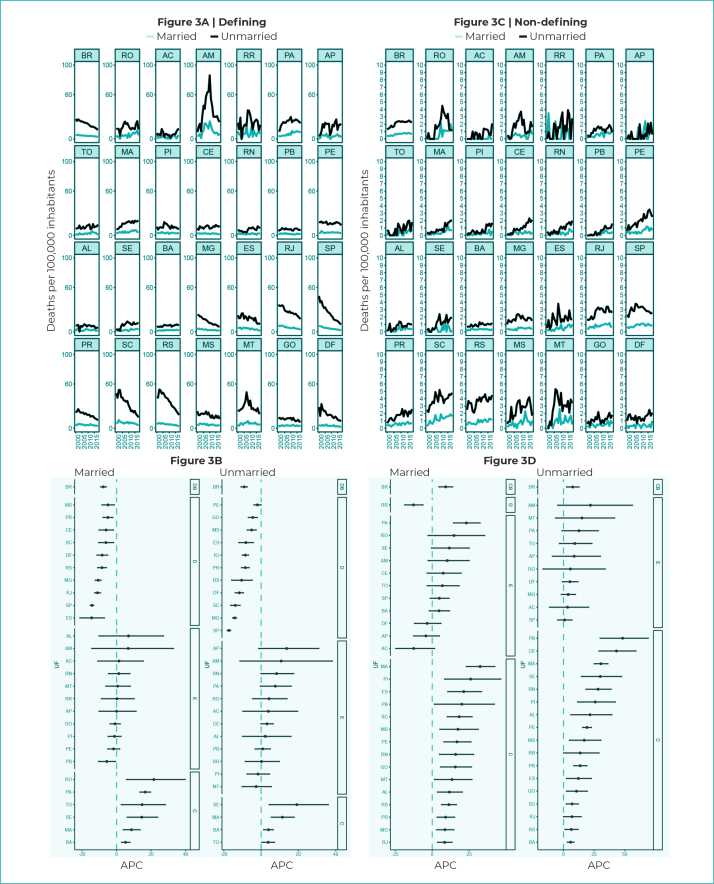
- Mortality rates and 95% confidence intervals with trends in HIV/AIDS-defining and non-HIV/AIDS defining illnesses, according to marital status, Federative Units and Brazil, 2000-2018

Legend: DB - decreasing trend in Brazil; SB - stationary trend in Brazil; IB - increasing trend in Brazil; D - decreasing trend; S - stationary trend; I - increasing trend; APC - Annual Percentage Change.

Mortality rates due to defining illnesses and according to marital status were higher among unmarried individuals, throughout the period (from 27.0 deaths in 2000 to 12.4 deaths/100,000 inhabitants in 2018) (Figure 3A). In the state of Amazonas, rates due to defining illnesses were higher among unmarried individuals (from 9.1 deaths in 2000 to 22.9 deaths/100,000 inhabitants in 2018), with oscillations over the period. The states of Roraima, Pará and Amapá also showed significant rates among unmarried individuals (Figure 3A). Mortality due to non-defining illnesses showed lower rates when compared to defining illnesses (Figure 3A).

In Brazil, there was a decreasing trend for defining illnesses among married (APC = -7.7%; 95%CI -9.3;-6.0) and unmarried individuals (APC = -9.1%; 95%CI -10.8;-7.5), while there was an upward trend in both categories when the trends in non-defining diseases were evaluated: for married individuals (APC = 9.1%; 95%CI 4.5;13.8) and for unmarried individuals (APC = 7.5%; 95%CI 2.5;-12.7) (Figure 3B).

**Figure 4:**
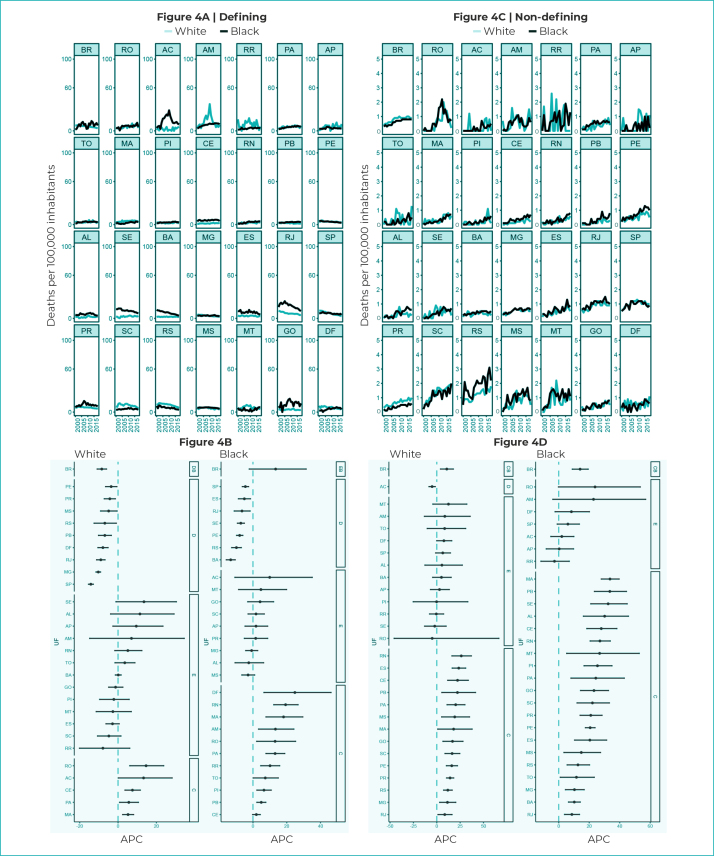
- Mortality rates and 95% confidence intervals with trends in HIV/AIDS-defining and non-HIV/AIDS defining illnesses, according to race/skin color, Federative Units and Brazil, 2000-2018

Legend: DB - decreasing trend in Brazil; SB - stationary trend in Brazil; IB - increasing trend in Brazil; D - decreasing trend; S - stationary trend; I - increasing trend; APC - Annual Percentage Change.

Mortality rates due to defining diseases in Brazil were higher when compared to non-defining diseases, also in the stratification by race/skin color (Figure 4A and 4D). Black people showed the highest rates for defining diseases, with the exception of the states of Amazonas, Roraima, Amapá, Maranhão, Santa Catarina, Rio Grande do Sul and Mato Grosso (Figure 4A). Mortality rates due to non-defining diseases were very close among White and Black races/skin color (Figure 4C).

Mortality due to HIV/AIDS-defining illnesses among White race/skin color showed an upward trend in the states of Rondônia (APC = 14.6%; 95%CI 6.1;23.8), Ceará (APC = 7.5; 95%CI 3.6;11.6), Acre (APC = 13.3%; 95%CI 0.1;28.2), Pará (APC = 5.6%; 95%CI 2.4;8.1) and Maranhão (APC = 5.2%; 95%CI 2.4;8.1) (Figure 4B).

With regard to Black race/skin color, there was an upward trend in most states, namely: Ceará (APC = 2.0%; 95%CI -0.2;4.3), Rio Grande do Norte (APC = 19.3%; 95%CI 12.3;26.7), Paraíba (APC = 4.9%; 95%CI 2.3;7.6), Federal District (APC = 24.8%; 95%CI 6.5;46.2), Amazonas (APC = 13.3%; 95%CI 3.3;24.2), Roraima (APC = 10.1%; 95%CI 4.6;15.8), Pará (APC = 13.1%; 95%CI 7.7;18.8), Tocantins (APC = 7.3%; 95%CI 0.1;15.0), Maranhão (APC = 18.1%; 95%CI 7.7;29.5) and Piauí (APC = 6.5%; 95%CI 2.3;10.8) (Figure 4B). Mortality due to non-defining diseases showed an upward trend, either among individuals of White race/skin color, or among those of Black race/skin color (Figure 4C).

## DISCUSSION

This study showed that the general mortality rates due to HIV/AIDS-defining illnesses are higher when compared to non-HIV/AIDS defining illnesses, regarding the analysis of the years between 2000 and 2018. According to sex, the rates for defining diseases were higher among males, compared to the female population.

In the observed period, mortality due to HIV/AIDS-defining illnesses showed a decreasing trend in the states of the South and Southeast regions, and upward and stationary trends in the North and Northeast regions, indicating the need for different measures aimed at controlling mortality due to the disease in these regions.^5^ ^,^ ^13^ ^-^ ^15^ Inequality in the regional distribution of services is likely to be contributing to this scenario.^4^

Nevertheless, non-HIV/AIDS defining illnesses showed an increasing trend in most states, when the general population and by sex were taken into consideration. The same pattern was found in other studies that pointed to an increase in deaths from non-defining illnesses.^2^ ^,^ ^3^ ^,^ ^16^

High mortality due to defining illnesses among males follows the pattern of mortality due to HIV/AIDS observed in the country and worldwide.^3^ ^,^ ^17^ ^-^ ^19^ However, females also need attention, because they have maintained an increasing trend in some states, when the defining illnesses are taken into consideration.

The oldest age groups, 30 to 59 and 60 years or over, showed higher rates of defining illnesses, while for non-defining illnesses, the age of 60 years or over showed more significant values. This scenario indicates that currently, mortality due to the disease can include individuals reaching advanced age who have acquired illnesses that are typical of HIV/AIDS, while the remaining people may be those who, after long-term ART use, suffered from the side effects of treatment and even from common conditions of older age.^3^ ^,^ ^16^ ^-^ ^21^

Regarding marital status, unmarried individuals showed higher rates of HIV/AIDS-defining illnesses. In addition, although mortality rates were higher among unmarried individuals, there was an upward trend, according to defining diseases, in the states of the Northeast region, and it could be seen an increasing trend, in most states, also among non-HIV/AIDS defining illnesses. High mortality rates among unmarried individuals were found in a study conducted in Papua, Indonesia, focusing on overall mortality, and the type of mortality was not detailed, whether due to defining diseases or not.^21^

Studies show that, depending on local culture, married individuals may be more vulnerable to HIV/AIDS, such as that observed in a rural area of South Africa, from 2000 to 2017, while in other locations the disease may be concentrated among unmarried individuals.^23^ ^,^ ^24^

The analysis based on race/skin color showed that mortality rates were higher among Black individuals in most states, according to defining diseases. There was also an upward trend in these rates, both for defining and non-defining illnesses. The highest proportion of the upward trend in non-defining illnesses was observed among Black individuals, while upward and stationary trends were found among White individuals, a result that may be linked to an ongoing increase in mortality due to non-defining illnesses among the population. Black population is in a greater social vulnerability^25^ ^-^ ^28^ and the change in their mortality patterns may occur at a slower rate than that observed in the population of White race/skin color.

A number of factors expose the population of Black race/skin color to mortality due to HIV/AIDS, such as unemployment, difficulty in accessing health care services and fear of seeking treatment due to discrimination.^26^

A study that analyzed the trend in mortality due to HIV/AIDS in Rio Grande do Sul and its capital, Porto Alegre, when checking mortality according to race/skin color, identified higher rates among Black and Brown individuals, between 2000 and 2011.^29^

This study has limitations related to possible incompleteness and inconsistencies in the data used. However, there was a significant improvement in SIM data resulting from the reduction in records of ill-defined causes of death. The analysis of comorbidities associated with HIV/AIDS may contribute to a better analysis of the dynamics of mortality due to HIV/AIDS and, consequently, a reduction in deaths from the disease.

Taking these results, it can be concluded that the findings of this research can contribute to the deepening of the analysis of mortality due to HIV/AIDS and thus, support the planning and management of public health actions. The existence of high mortality rates due to HIV/AIDS-defining diseases may indicate a scenario of inequalities in death from the disease. However, further studies are needed to deepen this analysis.
